# The case-area targeted rapid response strategy to control cholera in Haiti: a four-year implementation study

**DOI:** 10.1371/journal.pntd.0007263

**Published:** 2019-04-16

**Authors:** Stanislas Rebaudet, Gregory Bulit, Jean Gaudart, Edwige Michel, Pierre Gazin, Claudia Evers, Samuel Beaulieu, Aaron Aruna Abedi, Lindsay Osei, Robert Barrais, Katilla Pierre, Sandra Moore, Jacques Boncy, Paul Adrien, Florence Duperval Guillaume, Edouard Beigbeder, Renaud Piarroux

**Affiliations:** 1 Assistance Publique–Hôpitaux de Marseille (AP-HM), Marseille, France; 2 Hôpital Européen Marseille, Marseille, France; 3 Institut Pierre-Louis d’Epidémiologie et de Santé Publique, Sorbonne Université, INSERM, Paris, France; 4 United Nations Children's Fund, Haiti; 5 Aix Marseille Univ, IRD, INSERM, SESSTIM, Marseille, France; 6 Direction d’Epidémiologie de Laboratoire et de Recherche, Ministère de la Santé Publique et de la Population, Haiti; 7 Institut de Recherche pour le Développement (IRD), Marseille, France; 8 Direction de la Lutte contre la Maladie, Ministère de la Santé Publique, Kinshasa, Democratic Republic of the Congo; 9 Aix Marseille Univ, Marseille, France; 10 Laboratoire National de Santé Publique, Ministère de la Santé Publique et de la Population, Haiti; 11 Ministère de la Santé Publique et de la Population, former Minister, Delmas, Haiti; 12 Sorbonne Université, INSERM, Institut Pierre-Louis d’Epidémiologie et de Santé Publique, AP-HP, Hôpital Pitié-Salpêtrière, Paris, France; Johns Hopkins University Bloomberg School of Public Health, UNITED STATES

## Abstract

**Background:**

In October 2010, Haiti was struck by a large-scale cholera epidemic. The Haitian government, UNICEF and other international partners launched an unprecedented nationwide alert-response strategy in July 2013. Coordinated NGOs recruited local rapid response mobile teams to conduct case-area targeted interventions (CATIs), including education sessions, household decontamination by chlorine spraying, and distribution of chlorine tablets. An innovative *red*-*orange*-*green* alert system was also established to monitor the epidemic at the communal scale on a weekly basis. Our study aimed to describe and evaluate the exhaustiveness, intensity and quality of the CATIs in response to cholera alerts in Haiti between July 2013 and June 2017.

**Methodology/principal findings:**

We analyzed the response to 7,856 weekly cholera alerts using routine surveillance data and severity criteria, which was based on the details of 31,306 notified CATIs. The odds of CATI response during the same week (exhaustiveness) and the number of complete CATIs in responded alerts (intensity and quality) were estimated using multivariate generalized linear mixed models and several covariates. CATIs were carried out significantly more often in response to *red* alerts (adjusted odds ratio (aOR) [95%-confidence interval, 95%-CI], 2.52 [2.22–2.87]) compared with *orange* alerts. Significantly more complete CATIs were carried out in response to *red* alerts compared with *orange* alerts (adjusted incidence ratio (aIR), 1.85 [1.73–1.99]). Over the course of the eight-semester study, we observed a significant improvement in the exhaustiveness (aOR, 1.43 [1.38–1.48] per semester) as well as the intensity and quality (aIR, 1.23 [1.2–1.25] per semester) of CATI responses, independently of funds available for the strategy. The odds of launching a CATI response significantly decreased with increased rainfall (aOR, 0.99 [0.97–1] per each accumulated cm). Response interventions were significantly heterogeneous between NGOs, communes and departments.

**Conclusions/significance:**

The implementation of a nationwide case-area targeted rapid response strategy to control cholera in Haiti was feasible albeit with certain obstacles. Such feedback from the field and ongoing impact studies will be very informative for actors and international donors involved in cholera control and elimination in Haiti and in other affected countries.

## Author summary

Cholera is a potentially deadly diarrheal disease caused by toxin-secreting strains of *Vibrio cholerae*. The bacterium can trigger severe epidemics in countries with limited access to potable water and sanitation. Hygiene promotion, proper sanitation and improved access to safe drinking water are essential to control cholera. However, very few reports have described and evaluated the implementation of such activities in the field. Since October 2010, Haiti has been affected by one of the most severe cholera epidemics of the past few decades. In this report, we describe and evaluate the implementation of the original nationwide case-area targeted interventions (CATIs) response strategy, which was launched in July 2013 after years of insufficient response coordination and diminishing efforts to control cholera outbreaks in affected communities. Rapid implementation of education sessions, household decontamination, soap distribution and water chlorination in affected communities proved challenging but possible, even in a mountainous and decentralized country such as Haiti with tens of thousands of suspected cholera cases per year. Evaluation of the impact of the CATI-based strategy on the cholera epidemic is underway. Together with other components of a multi-sectoral approach, this rapid response strategy appears to be critical to eventually eliminate cholera in Haiti.

## Introduction

Cholera was accidentally imported into Haiti in October 2010 [1,2]. The country has consequently experienced a massive epidemic, with a total of 819,899 suspected cases and 9,791 cholera-related deaths by January 26, 2019 according to the Haitian Ministry of Health (MOH, acronyms summarized in S1 Table) [3]. Following international recommendations [4], Haitian authorities and services, together with numerous international and non-governmental organizations (NGOs), have struggled to mitigate the death toll and case incidence by supporting both cholera treatment institutions as well as safe water, improved sanitation and hygiene practice (WaSH) efforts in affected communities [5–8]. Cholera incidence gradually receded in 2011–2012, with alternating troughs and peaks influenced by seasonal rainfall [9]. Although Haiti was considered the country most affected by cholera worldwide [10], emergency funds for cholera declined and most organizations eventually interrupted or drastically reduced activities in 2012 [7,11]. During February 2013, the Haitian government, Pan American Health Organization (PAHO), United Nations Children's Fund (UNICEF) and the Centers for Disease Control and Prevention (CDC) launched an ambitious National Plan for the Elimination of Cholera in Haiti 2013–2022 [12]. Over $1.5 billion USD of the total $2.2 billion USD was designated to invest in Haitian water and sanitation infrastructures, as only 68% of households obtained drinking water from improved sources, 26% of households had access to improved sanitation facilities and 34% of households had water and soap available for handwashing [13]. Two pilot oral cholera vaccine (OCV) campaigns were carried out in 2012, vaccinating approximately 100,000 people in both rural and urban settings [14,15]. Two additional campaigns were planned for 2013 [16].

The elimination plan also intended to improve surveillance activities and ensure adequate outbreak response [12]. To interrupt local cholera outbreaks at an early stage, UNICEF supported the MOH and the Haitian National Directorate for Water and Sanitation (DINEPA) to launch a nationwide coordinated cholera alert-response strategy in July 2013. Analogous to forest fire management [17,18], this program aimed to rapidly detect local cholera outbreaks and send response teams to affected communities. Case-area targeted interventions (CATIs) involved detection of additional cases, house decontamination, awareness and health education concerning risk factors and methods of cholera prevention and management, soap distribution, and water chlorination at the household level or directly at water sources. At the central level, the epidemic was monitored using a simple and unique tri-color cholera alert system, which is described in detail in a preprint manuscript [19].

Based on the identified risk factors and the growing evidence of WaSH efficiency to control cholera [20–23] and diarrhea in developing countries or during humanitarian crises [24–27], hygiene promotion and improved access to safe drinking water have long been recommended to control cholera transmission [4, 28–30]. Interventions targeting the residential area of cholera cases and nearby neighbors appear important to control cholera outbreaks, as cholera risk has been shown to increase among neighbors living within a few dozen meters of cases during the first week following disease onset [31,32]. A recent modeling study has also supported the potential impact of early CATIs in response to cholera outbreaks [33]. However, feedback from the field has been scarce. Reported activities have usually been implemented at a local level, during short time periods, and described with few details [7,8, 29,34–47].

The aim of the present study was to describe and evaluate the implementation of CATIs to control cholera outbreaks in Haiti using output indicators [48] from July 2013 (epidemiological week 27) to June 2017 (week 26). Evaluation of the effectiveness and impact [48] of this strategy is outside the scope of the current paper, and dedicated studies are underway.

## Methods

### Rapid case-area targeted response interventions (CATIs) to control cholera outbreaks

The national alert-response strategy to control cholera in Haiti was launched in July 2013 to complement the multi-sectoral national plan for the elimination of cholera in Haiti 2013–2022 [12], which includes long-term WaSH infrastructure, medical care and OCV. The alert-response strategy aims to improve key aspects of infectious disease control in the country (Table 1 and S1 Fig): coordination of cholera control activities; epidemiologic surveillance of cholera; cholera prevention in the most vulnerable areas; and most importantly prompt and exhaustive case-area targeted response interventions (CATIs). To establish CATI teams, UNICEF established partnership with at least one WaSH NGO for each of the 10 administrative departments (S1 Table), which hired rapid response mobile teams comprised of local Haitian staff. They were encouraged to work in close collaboration with departmental health directorates and cholera treatment centers to obtain and share epidemiological cholera data and outbreak rumors on a daily basis (S1 Fig). Mobile teams were requested to respond to every suspected cholera case or death and every plausible rumor via CATI at the affected household and neighbors within 48 hours, based on the average cholera incubation period [49]. The teams were nevertheless encouraged to respond the same day if possible. In case of several concomitant outbreaks, mobile teams were asked to prioritize the most-affected areas. The response intervention methodology, which was established with the MOH and partners [50], is described in Table 1. House decontamination by chlorine spraying of latrines and other potentially contaminated surfaces was proposed to visited households, although the efficacy and impact of this method have never been established [30], and are likely limited to a few hours due to short-term vibrio survival [51]. CATIs were prospectively reported to UNICEF by mobile teams using standardized online Google spreadsheets. A few other organizations implementing CATIs and funded by other agencies also joined the strategy and reported their activities to UNICEF (S1 Table).

**Table 1:** Activities included in the national alert-response strategy to control cholera in Haiti and core methodology of case-area targeted response interventions.

Activities included in the national alert-response strategy to control cholera in Haiti		Actors
Improve coordination		
	of activities implemented by national, international, governmental and non-governmental partners involved in cholera control	MOH, DINEPA, UNICEF, PAHO, NGOs
Improve cholera surveillance		
	in the community and in cholera treatment institutions monitoring of outbreaks via an alert detection system at the central level	MOH, UNICEF, PAHO, NGOs
Case-area targeted response interventions		
Triggers	Every suspected case reported in a treatment center Rumors of cholera outbreaks	
Deadline	Max. 48 hours after case admission to a treatment center	
ore activities	Surveillance: verification of data in register books; identification of affected sites and neighborhoods Field investigations: extent of outbreak, outbreak origin, aggravating factors, contacts and suspected cases Visits to affected families and neighbors (minimum five households depending on the local geography) ^a^ Decontamination by chlorine spraying of latrines and other potentially contaminated surfaces ^a^ Education sessions about cholera transmission modes and methods of prevention and initial care ^a^ Distribution of one cholera kit per household: five soaps, five sachets of oral rehydration salts, and approximately 115 chlorine tablets (80 or 150 Aquatabs 33 mg in urban settings in rural areas, respectively) ^a^ Establishment of manual bucket chlorination at drinking water sources during one or more weeks when possible ^a^ Repair and extra-chlorination of water supply systems when possible ^a^	Response teams of WaSH NGO ^b^ (mainly contracted by UNICEF), MOH Rapid response teams (EMIRAs)
Additional medical activities	Primary care of community cases Chemoprophylaxis of close contacts with one dose of doxycycline (300 mg) for non-pregnant adults only Nursing support to cholera treatment institutions	EMIRAs Medical NGOs ^b^
Cholera prevention		
	in the most vulnerable areas Mass education sessions, communication for development (C4D) Rehabilitation or installation of water supply infrastructures Oral cholera vaccine campaigns	MOH, DINEPA UNICEF, PAHO NGOs

^a^ activities analyzed in the study

^b^ see S1 Table for a list of NGOs

DINEPA, National Directorate for Water and Sanitation; EMIRA, MOH departmental rapid response mobile teams; MOH, Ministry of Health (*Ministère de la Santé Publique et de la Population*); NGO, non-governmental organization; PAHO, Pan American Health Organization; UNICEF, United Nations Children's Fund; WaSH, water sanitation and hygiene promotion

To bolster institutional response capacities, UNICEF and the World Bank also provided additional material, funds and human resources to the MOH. The MOH established its own departmental response teams (EMIRAs, *Equipes Mobiles d’Intervention RApide*) on March 2014 to conduct CATIs and additional medical activities (Table 1), such as chemoprophylaxis of contacts living in the same house as cholera cases with one dose of doxycycline (300 mg) for non-pregnant adults only [4,52,53]. ECHO (European Commission Humanitarian Office) and PAHO (Pan American Health Organization) also contracted medical NGOs with terms of reference similar to that of the EMIRAs (Tables 1 and S1). In contrast to WaSH teams supported by UNICEF, reporting of EMIRA and mobile medical team activities was not systematic.

Field interventions involving both WaSH/medical and governmental/non-governmental staff were strongly encouraged and were carried out frequently. WaSH NGOs and EMIRAs typically formed common mixed mobile teams implementing core and supplemental medical activities (Table 1). Mobile teams were asked to repeat response interventions in the community until every suspected cholera case was addressed. In 2015, they were also requested to conduct surveys at targeted households to assess water treatment two weeks after the initial response intervention. These interventions are demonstrated in a short online video [54].

### Data collection

#### Cholera alerts and case-area targeted interventions

The cholera alert system has been detailed in a preprint article [19]. Alerts are listed in S1 Database, and they were computed for each of the 140 administrative communes of Haiti (S1 Fig) and the 209 weeks between July 2013 and June 2017, based on anonymized consolidated databases of institutional suspected cholera cases (acute watery diarrhea with or without vomiting, irrespective of patient age), cholera deaths and stool culture results of the MOH, and criteria listed in S2 Table. Reports of the CATIs carried out by mobile WaSH teams included date, location (*i*.*e*., commune, communal section, locality) and activities. We considered a CATI was complete if the corresponding report included a precise location, an education session, house decontamination, and either distribution of chlorine tablets, establishment of manual bucket chlorination at drinking water sources, or repair and extra-chlorination of water supply systems. The number of incomplete and complete CATIs are listed in S1 Database. For each CATI, mobile teams mentioned the institution that notified responded cases so that it was possible to link CATIs with cholera alerts by administrative commune and by week, even if CATIs were sometimes carried out in communes different from that where patients were treated. Unfortunately, targeted interventions performed by EMIRAs and medical NGO mobile teams could not be exhaustively quantified and described, although most WaSH interventions involved medical and governmental staff. Notably, exhaustive information concerning the use of doxycycline chemoprophylaxis was not available for analysis.

#### Covariates

To assess whether mobile teams prioritized more severe outbreaks, we distinguished *red* and *orange* alerts. To analyze the variation in CATI implementation in response to cholera alerts over time, we divided the four-year study period into eight semesters (first and last 26 weeks of every year). As response implementation may have been heterogeneous between NGOs, we identified the NGO responsible for response to alerts every week in every commune, using information provided by UNICEF and intervention reports. NGOs responsible for outbreak response changed over time, and NGOs could be responsible for communes in several departments at the same time (S1 Table). To assess the effect of weekly available funds on CATI implementation, we distributed all UNICEF disbursements for NGO and MOH mobile teams, in cash or in response items (e.g., chlorine, soap, buckets, oral rehydration salts), over the course of the 209-week study period. We also obtained population estimates for all 140 administrative communes from the Haitian Institute of Statistics and Informatics [55]. Commune remoteness was estimated by calculating distances from the main town or village of each commune to the capital Port-au-Prince and to the capital of the local department, using the OpenStreetMap road network [56]. As terrain may hinder CATI implementation, we identified mainly mountainous communes from a map of agro-ecological zones provided by FAO [57]. We identified communes that received mass OCV between 2012 and 2017, as this may have influenced CATI efforts. Finally, satellite estimates of daily-accumulated rainfall (area-averaged TRMM_3B42_daily v7) were extracted from the National Oceanic and Atmospheric Administration (NOAA) website covering the entire surface of Haiti and the centroid of all 140 communes [58]. The main characteristics of the communes in Haiti are summarized in S1 Database.

### Ethics statement

The protocol was authorized by the Haiti MOH National Bioethics Comity (authorization number #1718–30).

### Data analysis

As the cholera alert system was launched to prospectively monitor the epidemic, we used cholera alerts as an outbreak proxy to retrospectively evaluate the implementation of the case-area targeted response strategy. Hence, we could detect the capacity of response teams to directly obtain epidemiological information from the community, treatment institutions and departmental health directorates (S1 Fig). We then assessed three output indicators of the CATIs in response to cholera alerts: exhaustiveness, intensity and quality.

#### Analysis of the exhaustiveness of CATIs in response to cholera alerts

In an initial analysis, response exhaustiveness was defined as the proportion of retrospective *red* or *orange* alerts that triggered a response by at least one targeted intervention, either complete or incomplete, during the same epidemiological week. To illustrate response exhaustiveness, we plotted and mapped the numbers of responded and non-responded alerts per week and per commune, respectively. We then assessed the effect of several covariates on response exhaustiveness: administrative commune in alert; administrative department of the commune; responsible NGO; alert level (*red* versus *orange*); linear variation in response implementation over the course of eight semesters; UNICEF disbursements for CATIs during the week of the alert; weekly accumulated rainfall in the commune; commune population; OCV campaign in the commune; distance from Port-au-Prince; distance from the department capital; and terrain type (mountainous or non-mountainous). We used generalized linear mixed models (GLMMs) with alert response (responded vs non-responded alert) as an independent variable and a binomial distribution (logistic model) (Eq 1) [59].

$$E\left[y_{ij}|\nu_{i},x_{ij},z_{ij}\right]=g^{-1}\left(x_{ij}^{'}\beta +z_{ij}^{'}\nu_{i}\right)$$ (Eq 1)

where *y* represents the outcome variable, *x* represents the vector of covariates with fixed effect *β*, *z* represents the vector of covariates with random effect *ν*; *E*[.] represents the expectation of the conditional distribution of the outcome variable given the fixed and random effects, and *g*(.) represents the canonical link (*i*.*e*., the logit function for binomial distributions or the log function for negative-binomial ones).

For the univariate analyses of communes, departments and NGOs, each covariate was modeled separately as a unique random effect. For the univariate analyses of other covariates, we systematically included communes nested within departments as a common random effect and NGOs as a second random effect in models where each covariate was modeled as a unique fixed effect variable. For the multivariate analysis, we included the fixed effect variables for which *p*-values were less than 0.25 [60], communes nested within departments as a common random effect, and NGOs as a second random effect. The models estimated the crude odds ratio (cOR) and adjusted odds ratio (aOR) of response to alerts as well as 95% confidence intervals (95%-CI) associated with each covariate. A *p*-value of less than 0.05 indicated statistical significance.

#### Analysis of the intensity and quality of the CATI response to cholera alerts

In a second analysis restricted to responded alerts, response intensity and quality was defined together as the incidence of complete targeted interventions carried out per alert during the same epidemiological week. Using GLMMs with the number of complete CATIs per alert as an independent variable and a negative-binomial distribution (Eq 1) [59], we applied the same analysis procedure as that applied for response exhaustiveness. Models estimated the crude incidence ratio (cIR) and adjusted incidence ratio (aIR) for complete CATIs in responded alerts and 95% confidence intervals (95%-CI) associated with each covariate.

#### Software

Data management was performed using Microsoft Excel for Mac v15.32. QGIS v3.0.3 [61] was used to calculate distance matrices and draw the map. Graph design and statistical analyses were performed using R Studio version 1.1.453 for Mac [62] with R version 3.4.2 for Mac [63] and the {ggplot2} [64] and {lme4} [65] packages.

## Results

### Brief description of the epidemic and the response strategy

**Fig 1:**
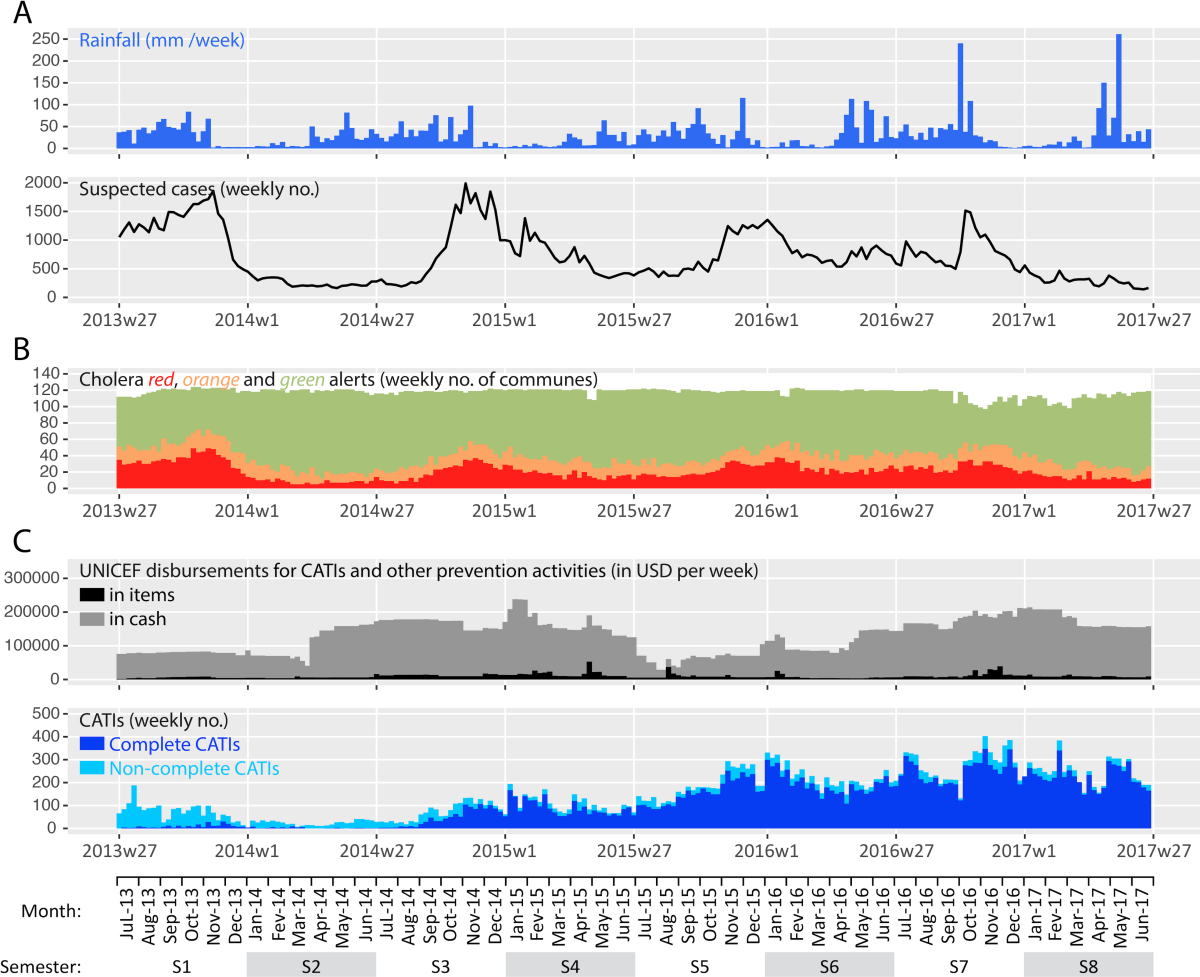
**Weekly evolution of accumulated rainfall and cases (Panel A), retrospective cholera alerts (Panel B), and implementation of the response strategy by UNICEF (Panel C) from mid-2013 (week 27) to mid-2017 (week 26).** Cumulated rainfall data averaged over the entire country was obtained from NOAA. Suspected cholera case numbers were obtained from the routine surveillance databases provided by the MOH. Retrospective cholera alerts were computed for a preprint manuscript, based on cases, deaths and stool cultures positive for *Vibrio cholerae* O1 [19]. Details on UNICEF disbursements and rapid CATIs were provided by UNICEF (S1 Database).

Between the launch of the nationwide alert-response strategy in July 2013 (week 27) and the end of this 209-week study period in June 2017 (week 26), a total of 149,690 suspected cholera cases were recorded throughout Haiti (Fig 1 Panel A). As a result, a total of 7,856 cholera alerts were identified in the country, including 4,365 *red* alerts and 3,491 *orange* alerts (Fig 1 Panel B) [19]. Alerts exhibited a temporal evolution consistent with the dynamics of the epidemic (Fig 1 Panels A and B). Alert distribution was geographically heterogeneous, as *red* and *orange* alerts mainly clustered in the departments of Ouest (especially in Port-au-Prince Metropolitan Area), Centre and Artibonite (S3 Table).

During the same period, UNICEF disbursed $25.4 million USD to support CATIs implemented by WaSH NGOs and MOH mobile teams (EMIRAs) as well as $2.0 million USD in response items (*i*.*e*., chlorine, soaps, buckets, oral rehydration salts) (Fig 1 Panel C). UNICEF delivered 3.3 million soaps, 140 million Aquatabs (33 mg tablets) and 3.6 million oral rehydration salt sachets to UNICEF partner organizations. These disbursements were not continuous over the study period (Fig 1 Panel C), and they appeared driven by various factors as detailed in S1 Text.

In four years, a total of 31,306 CATIs in response to cholera cases were notified by UNICEF WaSH partners (Fig 1 Panel C). Mobile teams performed education sessions to a total of 2.9 million people, decontaminated 179,830 houses, distributed chlorine tablets to 757,693 households, distributed soaps to 593,494 households, and supplied chlorination at 2,282 water sources or networks. A total of 25,202 CATIs (81%) was thus classified as complete. Over the course of the four-year study period, the overall incidence of CATIs and complete CATIs increased considerably (Fig 1 Panel C).

### Exhaustiveness of CATIs in response to cholera alerts

Between July 2013 and June 2017, mobile WaSH teams reported 31,306 CATIs to control cholera throughout the country, of which 61% were conducted in communes in *red* alert and 14% were carried out in communes in *orange* alert (data no shown). The remaining CATIs targeted *green* alert communes with sporadic cases (12%), *green* alert communes with no cases (7%) or communes with no data (6%).

**Fig 2:**
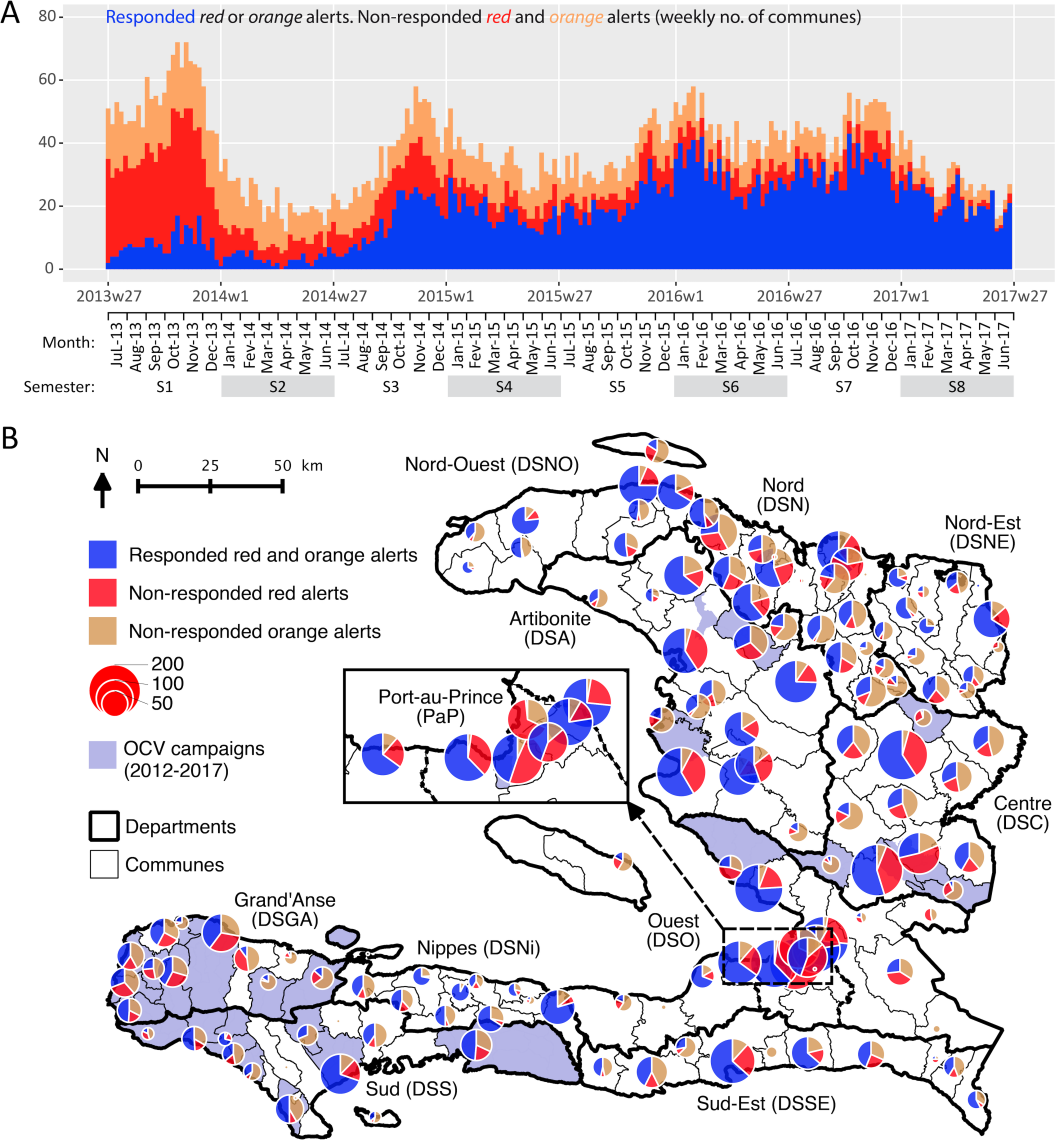
**Case-area targeted interventions (CATIs) in response to cholera alerts during the same week, from July 2013 to June 2017: weekly number (Panel A) and commune number (Panel B) of responded and non-responded *red* and *orange* alerts.** Retrospective cholera alerts were computed based on case numbers, death numbers and stool cultures positive for *Vibrio cholerae* O1 [19]. CATI records were provided by UNICEF. Oral cholera vaccine (OCV) records were provided by the MOH. The map was created using QGIS v3.0.3.

Between July 2013 and June 2017, mobile WaSH teams responded to 49% (3,824) of the 7,856 alerts during the same week. This proportion increased from 15% to 75% between the first and last semester of the four-year study period (Fig 2 Panel A, S3 Table, S2 Fig Panel A). Overall, the proportion of responded alerts appeared better for *red* alerts (58%) than for *orange* (37%) alerts (S3 Table and S2 Fig Panel A). The proportion of responded alerts appeared very heterogeneous between communes (Fig 2 Panel B). It ranged from 33% to 63% between departments. It ranged from 6% to 90% between NGOs (S3 Table and S2 Fig Panel A).

Using multivariate GLMMs, the odds of launching a CATI in response to cholera alerts (exhaustiveness) appeared significantly influenced by the commune, the department and the responsible NGO (common *p*-value of random effects < 0.01) (Table 2). Exhaustiveness of CATI response was significantly higher for *red* alerts than for *orange* alerts (adjusted odds ratio (aOR), 2.52 [2.22–2.86]; *p*-value < 0.0001). Exhaustiveness of CATI response significantly increased over the course of the study period (aOR, 1.43 [1.39–1.48] per semester; *p*-value < 0.0001) (Table 2), significantly decreased with accumulated rainfall (aOR, 0.99 [0.97–1] per each accumulated cm; *p*-value < 0.05), and tended to decrease when the alert was far from the department capital (aOR, 0.94 [0.88–1] per 10 km; *p*-value, 0.06). However, response exhaustiveness was not linearly dependent on UNICEF disbursements for CATIs. Response exhaustiveness was not significantly lower when the alert was far from Port-au-Prince, in a more populated commune, or in a mountainous commune. Finally, we found that response exhaustiveness was not significantly influenced by a previous OCV campaign (Table 2).

**Table 2:** Exhaustiveness of case-area targeted interventions (CATIs) in response to cholera alerts from July 2013 to June 2017: Factors associated with the odds of CATI response to alerts (logistic mixed models).

	*Red* and *orange* alerts		Univariate analysis ^b^^c^		Multivariate analysis ^d^	
	Responded	Non-responded	cOR [95%-CI]	*p*-value	aOR [95%-CI]	*p*-value
Number of alerts (%)	3824 (49%)	4032 (51%)				
Commune				<0.0001 ^b^		
Department ^a^				0.79 ^b^		
NGO responsible for CATI ^a^				0.3 ^b^		
Commune, department and NGO random effects ^d^						<0.01
Alert level, *red* vs. *orange* ^a^			2.22 [1.97–2.50]	<0.0001 ^c^	2.52 [2.22–2.86]	<0.0001
Semester since mid-2013 ^a^			1.42 [1.37–1.46]	<0.0001 ^c^	1.43 [1.38–1.48]	<0.0001
Weekly UNICEF disbursements for CATIs, mean (SD; $10,000 USD)	12.9 (4.7)	12 (4.7)	1.06 [1.05–1.07]	<0.0001 ^c^	1.01 [1.00–1.02]	0.22
Weekly accumulated rainfall in the commune, mean (SD; cm)	12.3 (14.2)	6.4 (9.6)	0.99 [0.98–1.00]	0.24 ^c^	0.99 [0.97–1]	<0.05
Population of the commune, mean (SD; 10,000 inhabitants)	12.3 (14.2)	6.4 (9.6)	1.03 [0.68–1.81]	<0.01 ^c^	1.01 [1–1.03]	0.11
OCV in the commune before or during the study period, number (%)	935 (24%)	1091 (27%)	1.11 [0.68–1.81]	0.67 ^c^	ND	ND
Distance from the capital Port-au-Prince, mean (SD; 10 km)	14.5 (8.1)	16.8 (7.8)	1.02 [0.97–1.08]	0.44 ^c^	ND	ND
Distance from the department capital, mean (SD; 10 km)	3.4 (2.7)	4.3 (3)	0.92 [0.86–0.98]	<0.01 ^c^	0.94 [0.88–1]	0.06
Mountainous commune, no. of alerts (%)	1456 (38%)	1646 (41%)	1.01 [0.68–1.50]	0.96 ^c^	ND	ND

Comparisons between responded and non-responded alerts were estimated using generalized linear mixed models with a binomial distribution.

^a^ Response rates for each class are summarized in S3 Table provided as supplementary information

^b^ For each of these univariate analyses, communes, departments or NGOs was modeled as a unique random effect variable.

^c^ For these univariate analyses, communes, departments and NGOs were modeled as random effect variables, with communes nested within departments. Models provided a common *P*-value for both random effects.

^d^ For the multivariate analysis, the model included communes, departments and NGOs as random effect variables, with communes nested within departments, and all fixed variables for which univariate *P-*value was <0.25 The model provided a common *P*-value for random effect variables.

SD, standard deviation; cOR, crude odds ratio; aOR, adjusted odds ratio; 95%-CI, 95% confidence interval; ND, no data (variables not included in the multivariate analysis).

### Intensity and quality of CATIs in response to cholera alerts

In each of the 3,824 *red* or *orange* responded alerts, an average of 545 persons (standard deviation (SD), 1,551) were reached by education sessions, 37 houses (SD, 68) were decontaminated by chlorine spraying, 141 families (SD, 579) received chlorine tablets for household water treatment, and an average of 0.4 water sources (SD, 2.6) were chlorinated during the same week (S3 Table). Overall, responded alerts thus received an average of 5.1 complete targeted interventions (SD, 7.8) during the same week. The mean number of complete CATIs per responded alert appeared better for *red* (6.4 [SD, 8.8]) than for *orange* (2.7 [4.4]) alerts (S3 Table and S2 Fig Panel B). This number increased from 0.7 (SD, 1.7) to 7.8 (10.6) between the first and last semester of the four-year study period (S3 Table and S2 Fig Panel B). The mean number of complete CATIs per responded alert ranged from 2.8 (3.1) to 10.4 (12.8) between departments and from 0 (0) to 11 (11.2) between NGOs (S3 Table and S2 Fig Panel B).

Using multivariate GLMMs, the incidence of complete CATIs conducted in responded alerts appeared significantly influenced by the commune, the department and the responsible NGO (common *p*-value of random effects < 0.001) (Table 3). The intensity and quality of CATI response was significantly higher for *red* alerts than for *orange* alerts (adjusted incidence ratio (aIR), 1.85 [1.72–1.98]; *p*-value < 0.0001). Response intensity and quality significantly increased over the course of the study period (aIR, 1.22 [1.20–1.25] per semester; *p*-value < 0.0001). Furthermore, response intensity and quality was greater in more populated communes (aIR, 1.02 [1.01–1.02] per 10,000 inhab.; *p*-value <0.0001) (Table 3). However, the incidence of complete CATIs in responded alerts was not significantly influenced by accumulated rainfall, UNICEF disbursements for CATIs, previous OCV campaigns, distance from Port-au-Prince or department capital, or mountainous terrain (Table 3).

**Table 3:** Intensity and quality of case-area targeted interventions (CATIs) in response to cholera alerts from July 2013 to June 2017: Factors associated with the incidence of complete CATIs per responded alert (negative-binomial mixed models).

	Univariate analysis ^b^^c^		Multivariate analysis ^d^	
	cIR [95%-CI]	*p*-value	aIR [95%-CI]	*p*-value
Mean number of complete CATIs per responded alert, 5.1 (SD, 7.8)				
Commune		<0.0001 ^b^		
Department ^a^		<0.0001 ^b^		
NGO responsible for CATI ^a^		0.98 ^b^		
Commune, department and NGO random effects ^d^				<0.001
Alert level, *red* vs. *orange* ^a^	1.72 [1.60–1.85]	<0.0001 ^c^	1.85 [1.72–1.98]	<0.0001
Semester since mid-2013 ^a^	1.21 [1.18–1.23]	<0.0001 ^c^	1.22 [1.20–1.25]	<0.0001
Weekly UNICEF disbursements for CATIs, mean (SD; $10,000 USD)	1.03 [1.02–1.03]	<0.0001 ^c^	1.00 [0.99–1.01]	0.84
Weekly accumulated rainfall in the commune, mean (SD; cm)	1.00 [0.99–1.01]	0.92 ^c^	ND	ND
Population of the commune, mean (SD; 10,000 inhab.)	1.02 [1.01–1.03]	<0.0001 ^c^	1.02 [1.01–1.02]	<0.0001
OCV in the commune before or during the study period, number (%)	1.18 [0.92–1.51]	0.18 ^c^	0.99 [0.81–1.20]	0.91
Distance from the capital Port-au-Prince, mean (SD; 10 km)	1.01 [0.98–1.04]	0.4 ^c^	ND	ND
Distance from the department capital, mean (SD; 10 km)	0.96 [0.93–1.00]	<0.05 ^c^	1.00 [0.97–1.02]	0.77
Mountainous commune, number of alerts (%)	1.05 [0.85–1.29]	0.65 ^c^	ND	ND

Comparison of the number of complete CATIs per responded alert was estimated using generalized linear mixed models with a negative-binomial distribution.

^a^ Number of complete CATIs for each class are summarized in S3 Table provided as supplementary information.

^b^ For each of these univariate analyses, communes, departments or NGOs was modeled as unique random effect variables.

^c^ For these univariate analyses, communes, departments and NGOs were modeled as random effect variables, with communes nested within departments. Models provided a common p-value for both random effects.

^d^ For all multivariate analysis, the model included communes, departments and NGOs as random effect variables, with communes nested within departments, and all fixed variables for which univariate p-value was <0.25 The model provided a common p-value for random effect variables.

SD, standard deviation; cIR, crude incidence ratio; aIR, adjusted incidence ratio; 95%-CI, 95% confidence interval; ND, no data (variables not included in the multivariate analysis).

## Discussion

Our analysis of 31,306 CATIs carried out by mobile WaSH teams between July 2013 and June 2017 shows that implementation of the first nationwide coordinated alert-response strategy to control cholera in Haiti was feasible at an annual cost of less than $1 USD per inhabitant. Response exhaustiveness, intensity and quality were initially insufficient but markedly improved over the course of the study period, with 75% of alerts receiving a response in the same week during the last semester of this four-year study. This amelioration was independent of available funds, which suggests that a significant buffer period was necessary to establish coordination between response partners and the MOH, to organize and commit mobile teams, and to secure administrative support for the strategy, as described in S1 Text. Response interventions were significantly heterogeneous between contracted NGOs, which was likely due to disparities in NGO engagement and capacity to coordinate activities, notably with peripheral health authorities. Response interventions were also heterogeneous between communes and departments, which reflects logistic obstacles to reach cholera outbreaks in remote areas, as confirmed by the significantly lower odds of CATI response during heavy rainfall. Response to alerts was however not significantly hampered by the distance between the affected commune and Port-au-Prince, probably thanks to the decentralized response capacity at the department level. Finally, the odds of response were significantly higher for *red* alerts than *orange* alerts, and significantly more complete CATIs were conducted in response to *red* alerts affecting the more populated communes, thus suggesting that response teams prioritized more severe outbreaks, as requested.

To monitor the response strategy, we chose to use alerts that were retrospectively computed based on consolidated surveillance databases [19]. Cholera alerts proved to be a practical and original indicator, albeit with several limits. Alerts could not be used to accurately assess response promptness because the weekly alert time scale largely exceeded the 48-hour intervention deadline that mobile teams were requested to respect. As the quality of reporting appeared heterogeneous especially at the beginning of the study period, we could not analyze CATI details, such as the exact quantities of distributed items, nor medical activities carried out by MOH mobile teams and medical NGOs, such as active case finding and chemoprophylaxis. Several other organizations also operated in community cholera prevention during the study period, such as Brigada Médica Cubana, Médecins sans Frontières–Netherlands, Gheskio, Zanmi Lasante, and Canadian Red Cross. However, their cholera field response activities appeared limited in comparison with the 31,306 CATIs included in the present study (field investigation results). Finally, the results may have been biased by the fact that our statistical analyses did not really consider temporal and spatial autocorrelation of alerts and targeted interventions. Both aspects were however partially included in the mixed models, by assessing the covariates semester and remoteness of communes and departments. We therefore believe our analyses were relevant enough to assess the overall dynamics and key determinants of targeted intervention implementation.

Further evaluation is required to assess several additional outputs and outcomes of response interventions, including exact promptness (daily scale); exact geographic targeting; number of persons reached; education session methodology; quantity of distributed items; changes in knowledge, attitude and practice of targeted households concerning handwashing, defecation and water treatment [48,66,67]. Analysis of data from surveys conducted two weeks after the initial response intervention to assess water treatment in targeted households is ongoing. Considering the potential risk of bacterial drug resistance [52], the use of doxycycline chemoprophylaxis should be evaluated in detail. After three years of reactive use and regular monitoring of antibiotic resistance by the MOH, it seems that no resistant *Vibrio cholerae* clinical strains have been isolated in Haiti (unpublished data from the MOH).

This study was not designed to evaluate the effectiveness and impact of the response strategy. Pre-published complementary results suggest that this CATI strategy was significantly effective in mitigating and shortening cholera outbreaks between 2015 and 2017 in Centre Department [68]. Another similar study is underway for the entire country. In addition to the OCV campaigns that have targeted 10% of the population since 2012 [69], the slow progress achieved in water infrastructure provision [70], and the other prevention activities conducted by the MOH, DINEPA and other organizations, this national alert-response strategy may thus have contributed to reduce cholera incidence in 2014 and since 2017 [3].

The present evaluation thus shows that the unprecedented large-scale implementation of CATIs to control cholera is achievable and may cost less than $1 USD per inhabitant and per year. This strategy however requires continuous efforts to improve response exhaustiveness, intensity and quality. Uniform methodology, centralized coordination, decentralized response capacity, and sustained funding should be key elements of similar strategies. Recent preliminary results concerning CATI effectiveness in Haiti seem promising [68] and will help to optimize future elimination efforts. In Haiti, CATIs still constitute a core element of the 2016–2018 mid-term development of the national plan for cholera elimination [71] and the UN’s new approach to cholera in Haiti, which was adopted by the General Assembly in December 2016 [72]. On a global scale, rapid response has also become a key component of the new multi-sectoral approach promoted by the Global Task Force on Cholera Control to reduce cholera deaths by 90% and eliminate the disease in 20 countries by 2030 [73]. Together with the results of ongoing impact studies, these lessons learnt from the field will be very informative for actors and international donors involved in cholera control and elimination, both in Haiti and other countries affected by cholera outbreaks.

## Supporting information

S1 Table Main organizations involved in case-area targeted interventions. (PDF)

S1 Fig Organization of cholera surveillance and rapid response in Haiti. (PDF)

S1 Text Narrative of the epidemic and response dynamics. (PDF)

S2 Table Definitions of cholera alerts used in the study. (PDF)

S3 Table Response to alerts, CATI activities in responded alerts, number of complete CATIs for responded alerts. Difference between departments, NGOs, alert levels, and semesters over the course of the study.
(PDF)

S2 Fig **Response to cholera alerts by case-area targeted interventions during the same week, from July 2013 to June 2017**: responded and non-responded red and orange alerts (Panel A) and number of complete CATIs per responded alert (Panel B). Difference between alert levels, change over the course of the eight-semester study period, and difference between departments.
(PDF)

S4 Table List of the databases used in the study. (PDF)

S1 Database Databases used in the study. (XLSX)
