# Supplementary material for: The case-area targeted rapid response strategy to control cholera in Haiti: a four-year implementation study
Source: PLoS Negl Trop Dis. 2019 Apr 16;13(4):e0007263. doi: 10.1371/journal.pntd.0007263 (PMC6485755; doi:10.1371/journal.pntd.0007263)
Supplement: S1 Table — (PDF) [file pntd.0007263.s001.pdf]

S1 Table. Main organizations involved in case-area targeted interventions (CATIs).

Acronym	Name of organization	Zone of activity
Haitian governmental response teams		
EMIRA	Departmental rapid response mobile teams of the Ministry of Health	Every department since 2014
WASH (Water Sanitation and Hygiene promotion) non-governmental organizations (mainly contracted by UNICEF)		
ACF[ab]	Action Against Hunger	DSA and DSNO since 2013
ACTED[ab]		DSS since 2013, DSGA, DSA and DSO since 2014, DSC since 2016
Care[ab]		DSGA in 2013
FONDEFH[ab]	*Fondation pour le développement et l'encadrement de la famille haïtienne*	DSN and DSNE in 2013
FRC[ab]	French Red Cross, works with Haitian Red Cross	DSO since 2013, DSA in 2015-2016
IFRC[ab]	International Federation of Red Cross and Red Crescent, works with Haitian Red Cross	DSO since 2017
Oxfam[ab]	Worked with OSAPO (*Oganizasyon Sante Popilè*) in 2013	DSN and DSNE since 2013, DSC since 2015
PLAN[ab]	Plan International	DSSE in 2013
RCG[a]	Red Cross Germany, works with Haitian Red Cross	DSO in 2013
SI[ab]	Solidarités International	DSNi since 2013, DSSE and DSO since 2014
SRC[a]	Swiss Red Cross	DSO in 2013
UNOPS[a]	United Nations Office for Project Services	DSS in 2013, DSO in 2014
ZL[ab]	Zanmi Lasante	DSC (part of the response strategy in 2013-2014)
Health organizations (contracted by other agencies or independent)		
GHESKIO[b]		Port-au-Prince Metropolitan Area in 2013-2014
IMC	International Medical Corps	DSN and DSNE since 2014
IOM	International Organization for Migration	DSO and DSA since 2014, DSSE in 2013
MDM	*Médecins du Monde* consortium (Belgium, France, Spain sections)	DSGA, DSNi and DSO since 2013, DSS, DSA and DSNO since 2014
MSF-H	*Médecins sans Frontières* – Netherlands	Entire country since 2010

[a] organizations for which interventions were included in the study
[b] organizations that received UNICEF funds via the alert-response strategy
Department abbreviations: DSA, Artibonite; DSC, Centre; DSGA, Grand'Anse; DSNi, Nippes; DSN, Nord; DSNE, Nord-Est; DSNO, Nord-Ouest; DSO, Ouest; DSS, Sud; and DSSE, Sud-Est.
