# Supplementary figures and images for: The case-area targeted rapid response strategy to control cholera in Haiti: a four-year implementation study
Source: PLoS Negl Trop Dis. 2019 Apr 16;13(4):e0007263. doi: 10.1371/journal.pntd.0007263 (PMC6485755; doi:10.1371/journal.pntd.0007263)

**S1 Figure. Organization of cholera surveillance and rapid response in Haiti.**

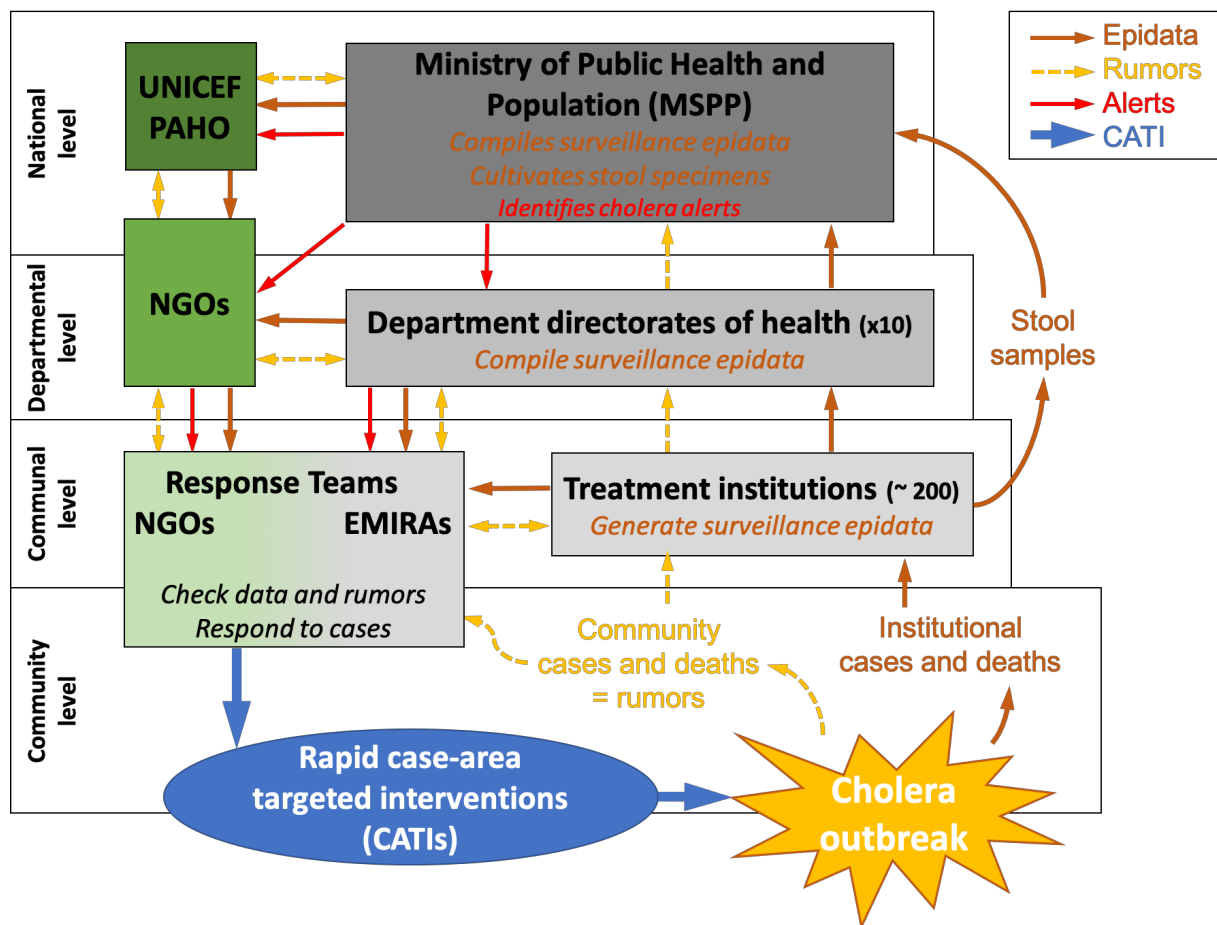

Supplement: S1 Fig — (PDF) [file pntd.0007263.s002.pdf]
