# Supplementary material for: The case-area targeted rapid response strategy to control cholera in Haiti: a four-year implementation study
Source: PLoS Negl Trop Dis. 2019 Apr 16;13(4):e0007263. doi: 10.1371/journal.pntd.0007263 (PMC6485755; doi:10.1371/journal.pntd.0007263)
Supplement: S1 Text — (PDF) [file pntd.0007263.s003.pdf]

S1 Text. Narrative of the epidemic and response dynamics

The nationwide alert-response strategy against cholera in Haiti started during the 2013 rainy season. The case load slowly rose to 1,614 weekly suspected cases in November 2013 (Fig 1 Panel A). Cholera incidence then collapsed concomitantly with the dry season and approximately 50 response interventions weekly. An unprecedented 38-week period with under 500 notified cases weekly (including 23 weeks under 250 cases) extended until late September 2014, despite regular precipitation from March onward and only few notified CATIs regardless of the increase in available funds. This temporary reduction in field interventions was consecutive to contract handovers of several partner organizations and a severe but under-reported chikungunya epidemic that affected a large proportion of NGO staff and field workers [1]. An abrupt increase in cholera incidence was then observed in September, which peaked at 1,990 cases at 2014w45. This severe outbreak primarily affected the Port-au-Prince Metropolitan Area and probably involved the contamination of a main water supply pipe vandalized by gangs who were reselling water in nearby slums (results from unpublished field investigations). In response to this crisis in the capital that persisted throughout the 2014-2015 dry season, UNICEF advocated for additional funds from OCHA for mobile WaSH teams and intensified field interventions. Concomitantly, UNICEF reorganized the coordination of contracted NGOs in order to better harmonize the methodology of interventions. The strategy was further bolstered during the second semester of 2015 and during the first semester of 2016 thanks to a $8.0 million USD loan from the UN. In 2015 and 2016, cholera case numbers oscillated between 500 and 1,500 per week with seasonal fluctuations, notably following hurricane Matthew that struck the departments of South and Grand'Anse in October 2016. Through additional funds, a minimum of 60 mobile WaSH NGO teams supported the 13 mobile teams of the MOH (EMIRAs) to maintain a high volume of field interventions until the end of the study period in June 2017. Cholera incidence then exhibited a continuous decrease and remained below 500 cases per week in 2017, although exceptional precipitation levels were recorded in April and May (Fig 1).

1. Staples JE, Fischer M. Chikungunya Virus in the Americas — What a Vectorborne Pathogen Can Do. N Engl J Med. 2014;371: 887–889. doi:10.1056/NEJMp1407698
