# Supplementary material for: The case-area targeted rapid response strategy to control cholera in Haiti: a four-year implementation study
Source: PLoS Negl Trop Dis. 2019 Apr 16;13(4):e0007263. doi: 10.1371/journal.pntd.0007263 (PMC6485755; doi:10.1371/journal.pntd.0007263)
Supplement: S2 Table — (PDF) [file pntd.0007263.s004.pdf]

S2 Table. Definitions of cholera alerts used in the study

Red alert	
	≥1 cholera-associated hospital or community death of an individual ≥ five years of age during the past seven days
and/or	≥10 suspected cholera cases aged ≥ five years during the past seven days
and/or	≥1 stool culture positive for *Vibrio cholerae* O1 at the LNSP
Orange alert	
	No red alert criteria
and	Twofold or more increase in suspected cases aged ≥ five years during the past seven days compared with the previous seven-day period
and/or	Red alert during the previous week
Green alert	
	No red or orange alert criteria for at least two weeks

Official cholera alert criteria used by the Directorate of Epidemiology Laboratory and Research (DELR) of the Haitian Ministry of Health

LNSP, National Laboratory of Public Health
