# Supplementary material for: The case-area targeted rapid response strategy to control cholera in Haiti: a four-year implementation study
Source: PLoS Negl Trop Dis. 2019 Apr 16;13(4):e0007263. doi: 10.1371/journal.pntd.0007263 (PMC6485755; doi:10.1371/journal.pntd.0007263)
Supplement: S3 Table — Difference between departments, NGOs, alert levels, and semesters over the course of the study. (PDF) [file pntd.0007263.s005.pdf]

S3 Table. Response to alerts, CATI activities in responded alerts, numbers of complete CATIs for responded alerts. Differences between departments, NGOs, alert levels, and semesters over the course of the study.

	All alerts	Responded alerts	Non-responded alerts	Education session in responded alerts	Decontamination by spraying in responded alerts	Distribution of chlorine tablets in responded alerts	Chlorination at water sources in responded alerts	Complete CATIs in responded alerts
	no.	no. (%)	no. (%)	mean no. of persons (SD)	mean no. of houses (SD)	mean no. of households (SD)	mean no. of sources (SD)	mean no. (SD)
**All alerts**	7856	3824 (49%)	4032 (51%)					
**Responded alerts**				545 (1551)	37 (68)	141 (579)	0.4 (2.6)	5.1 (7.8)
**Alert level**								
Red alerts	4365	2516 (58%)	1849 (42%)	683 (1842)	47 (76)	171 (663)	0.5 (3.1)	6.4 (8.8)
Orange alerts	3491	1308 (37%)	2183 (63%)	278 (631)	19 (44)	82 (361)	0.3 (1)	2.7 (4.4)
**Department**								
DSNO	560	329 (59%)	231 (41%)	417 (1724)	28 (31)	120 (1137)	0.8 (1.7)	2.8 (3.1)
DSN	1150	463 (40%)	687 (60%)	639 (1508)	25 (31)	82 (386)	0.1 (0.8)	3.5 (4)
DSNE	423	206 (49%)	217 (51%)	635 (2162)	32 (42)	99 (283)	0.2 (1.2)	3.2 (3.4)
DSA	1297	704 (54%)	593 (46%)	404 (635)	21 (27)	170 (691)	0.4 (2.6)	3.2 (5.4)
DSC	1035	412 (40%)	623 (60%)	443 (2827)	30 (30)	116 (523)	1.2 (6.3)	5 (6.1)
DSO	1493	803 (54%)	690 (46%)	773 (1491)	76 (123)	155 (257)	0.1 (1)	10.4 (12.8)
DSNI	349	220 (63%)	129 (37%)	173 (354)	16 (25)	68 (321)	0.3 (0.9)	4.2 (4.5)
DSSE	500	258 (52%)	242 (48%)	216 (354)	27 (41)	35 (41)	0.1 (0.4)	4.8 (4.9)
DSS	475	239 (50%)	236 (50%)	1152 (1436)	53 (71)	394 (838)	1.4 (2.3)	4.1 (5.5)
DSGA	574	190 (33%)	384 (67%)	329 (563)	16 (23)	159 (548)	0.5 (1.4)	2.9 (3.6)
**Semester since mid-2013**								
S1 (Jul 2013 - Dec 2013)	1438	213 (15%)	1225 (85%)	1557 (4739)	6 (20)	726 (1782)	0.1 (0.6)	0.7 (1.7)
S2 (Jan 2014 - Jun 2014)	561	87 (16%)	474 (84%)	749 (1065)	3 (8)	820 (1907)	0.1 (0.4)	0.3 (1)
S3 (Jul 2014 - Dec 2014)	935	388 (41%)	547 (59%)	966 (2015)	23 (34)	124 (297)	0.9 (3.6)	2.3 (3)
S4 (Jan 2015 - Jun 2015)	896	456 (51%)	440 (49%)	929 (1752)	38 (63)	150 (303)	1 (6)	4.1 (7.3)
S5 (Jul 2015 - Dec 2015)	962	559 (58%)	403 (42%)	454 (581)	34 (41)	76 (148)	0.4 (1.3)	5.7 (8.4)
S6 (Jan 2016 - Jun 2016)	1145	762 (67%)	383 (33%)	261 (356)	33 (49)	56 (141)	0.3 (0.9)	5.5 (8.1)
S7 (Jul 2016 - Dec 2016)	1179	804 (68%)	375 (32%)	331 (745)	40 (55)	73 (241)	0.3 (1.4)	6.1 (6.7)
S8 (Jan 2017 - Jun 2017)	740	555 (75%)	185 (25%)	305 (454)	67 (130)	92 (159)	0.3 (1.5)	7.8 (10.6)

(continued)	All alerts	Responded alerts	Non-responded alerts	Education session in responded alerts	Decontamination by spraying in responded alerts	Distribution of chlorine tablets in responded alerts	Chlorination at water sources in responded alerts	Complete CATIs in responded alerts
		no. (%)	no. (%)	mean no. of persons (SD)	mean no. of houses (SD)	mean no. of households (SD)	mean no. of sources (SD)	mean no. (SD)
**NGO on duty for CATI**								
NGO #1	1602	828 (52%)	774 (48%)	388 (1163)	22 (28)	165 (956)	0.6 (2.6)	1.8 (2.5)
NGO #2	1298	758 (58%)	540 (42%)	778 (1316)	42 (63)	227 (564)	0.7 (1.9)	6.2 (7.1)
NGO #3	142	9 (6%)	133 (94%)	1427 (873)	0 (0)	1137 (1008)	0 (0)	0 (0)
NGO #4	657	383 (58%)	274 (42%)	474 (1006)	36 (75)	97 (168)	0 (0.2)	6 (8.2)
NGO #5	32	29 (91%)	3 (9%)	1256 (312)	3 (5)	194 (98)	0 (0)	0 (0)
NGO #6	39	35 (90%)	4 (10%)	527 (487)	74 (69)	107 (98)	0.2 (0.8)	11 (11.2)
NGO #7	280	17 (6%)	263 (94%)	3656 (3418)	0 (1)	997 (1187)	0.1 (0.2)	0.4 (0.7)
NGO #8	232	5 (2%)	227 (98%)	146 (135)	8 (7)	7 (7)	0 (0)	0.6 (0.5)
NGO #9	1752	943 (54%)	809 (46%)	456 (1342)	28 (33)	69 (253)	0.3 (1.1)	3.9 (3.9)
NGO #10	1194	727 (61%)	467 (39%)	375 (870)	65 (116)	94 (237)	0.1 (0.6)	9.6 (12.5)
NGO #11	108	31 (29%)	77 (71%)	2161 (3182)	1 (3)	109 (452)	0 (0.2)	0.2 (0.5)
NGO #12	510	59 (12%)	451 (88%)	1538 (7341)	26 (31)	317 (1277)	4.9 (16)	1 (0.9)
