# Supplementary material for: The case-area targeted rapid response strategy to control cholera in Haiti: a four-year implementation study
Source: PLoS Negl Trop Dis. 2019 Apr 16;13(4):e0007263. doi: 10.1371/journal.pntd.0007263 (PMC6485755; doi:10.1371/journal.pntd.0007263)

S2 Figure. Response to cholera alerts by case-area targeted interventions (CATIs) during the same week, from July 2013 to June 2017: responded and non-responded *red* and *orange* alerts (Panel A) and number of complete CATIs per responded alert (Panel B). Difference between alert levels, change over the course of the eight semesters of the study period, and difference between departments. In Panel A, stacked bar plots show the following: blue, the number of *red* or *orange* alerts that were responded to by CATI during the same week; red and orange, the number of *red* or *orange* alerts that were *not* responded to during the same week. Black dashes show the proportion of *red* or *orange* alerts that were responded to during the same week. In Panel B, dots show the number of complete CATIs per responded alert. Point ranges show the mean and standard deviation (SD) of complete CATIs per responded alerts. Department abbreviations: DSNO, Nord-Ouest; DSN, Nord; DSNE, Nord-Est; DSA, Artibonite; DSC, Centre; DSO, Ouest; DSNi, Nippes; DSSE, Sud-Est; DSS, Sud; DSGA, and Grand'Anse.

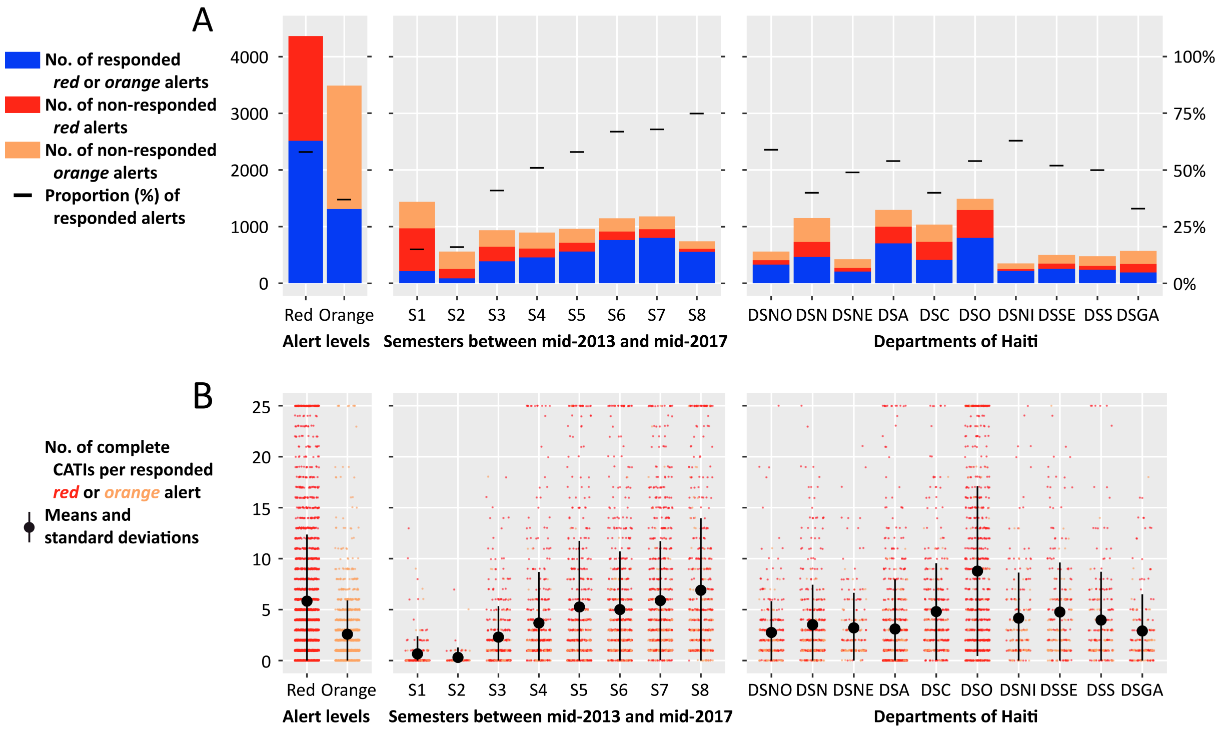

Supplement: S2 Fig — Response to cholera alerts by case-area targeted interventions during the same week, from July 2013 to June 2017: responded and non-responded red and orange alerts (Panel A) and number of complete CATIs per responded alert (Panel B). Difference between alert levels, change over the course of the eight-semester study period, and difference between departments. (PDF) [file pntd.0007263.s006.pdf]
