# Supplementary material for: The case-area targeted rapid response strategy to control cholera in Haiti: a four-year implementation study
Source: PLoS Negl Trop Dis. 2019 Apr 16;13(4):e0007263. doi: 10.1371/journal.pntd.0007263 (PMC6485755; doi:10.1371/journal.pntd.0007263)
Supplement: S4 Table — (PDF) [file pntd.0007263.s007.pdf]

## S4 Table. List of the databases used in the study

| Database | Row unit | Main variables of interest | Spatial scale | Time scale | Begin Date | End date | Database constitution | Data sources | Cleaning of data | Availability of database | Main potential sources of bias | Efforts to address potential sources of bias |
|---|---|---|---|---|---|---|---|---|---|---|---|---|
| Cholera alerts | Commune | Weekly cholera alert level retrospectively computed | Communes | Weeks | 2013 27th epi week | Ongoing | Authors | See dedicated publication[a] | No need | See S1 Database | See dedicated publication[a] | See dedicated publication[a] |
| Case-area targeted interventions (CATIs) and complete CATIs | Commune | Weekly number of CATIs and complete CATIs in each administrative commune | Communes | Weeks | 2013 27th epi week | Ongoing | UNICEF-Haiti | Intervention reports of response mobile teams | Regular prospective cleaning and quality-control of intervention reports using field information. | Upon approval by UNICEF-Haiti<br>See S1 Database | Under-reporting and misreporting of response interventions | Regular field investigations |
| Commune characteristics | Administrative Commune | Commune name, department, coordinates, population, accumulated rainfall, mountainous relief, distances to Port-au-Prince and to the department capital, start date of OCV campaigns | Communes | NA | NA | NA | Authors | See Methods | No need | See S1 Database | | |
| NGO responsible for CATI | Commune | Weekly ID of the main NGO responsible for CATI response | Communes | Weeks | 2013 27th epi week | Ongoing | Authors | Contracts between UNICEF and NGOs and intervention reports | No need | See S1 Database | | |

CATI, case-area targeted intervention; complete CATI, CATI report including a precise location, an education session, the decontamination of households, and either the distribution of chlorine tablets, the establishment of manual bucket chlorination at drinking water sources, or the repair and extra-chlorination of water supply systems

[a] Rebaudet S, Bulit G, Gaudart J, Michel E, Gazin P, Evers C, et al. The national alert-response strategy against cholera in Haiti: a four-year assessment of its implementation. bioRxiv. 2018; 259366. doi:10.1101/259366.
